# A Co-Expressed Natural Antisense RNA *FCER1A*-AS Controls IgE-Dependent Immunity by Promoting Expression of FcεRIα

**DOI:** 10.1128/spectrum.00733-23

**Published:** 2023-05-08

**Authors:** Ruo-yu Tang, Lan Yin, Liang Yao, Qing-Feng Zhang, Xiao-Ping Chen

**Affiliations:** a Department of Microbiology and Immunology, School of Medicine, Tongji University, Shanghai, China; Hubei University of Medicine

**Keywords:** antisense RNA, FcεRIα, *FCER1A*-AS, IgE, allergic reaction, *Schistosoma japonicum*

## Abstract

As the α-subunit of the high-affinity receptor for the Fc portion of immunoglobulin E (FcεRIα), FcεRIα plays a central role in IgE-mediated allergic disorders and in the immunity and immunopathology of some parasitic infections. FcεRIα is specifically expressed on basophils and mast cells, but the mechanism that controls FcεRIα expression in these cells is poorly understood. In this study, we found that the natural antisense transcript (NAT) of FcεRIα (*FCER1A*-AS) is co-expressed with the sense transcript (*FCER1A*-S) in both interleukin (IL)-3-induced FcεRIα-expressing cells and in the high FcεRIα-expressing cell line MC/9. When *FCER1A*-AS is selectively knocked down by the CRISPR/RfxCas13d (CasRx) approach in MC/9 cells, the expression of both *FCER1A*-S mRNA and proteins is markedly decreased. Furthermore, *FCER1A*-AS deficiency was also found to be associated with a lack of *FCER1A*-S expression *in vivo*. Correspondingly, homozygous mice deficient in *FCER1A*-AS demonstrated a similar phenotype to *FCER1A* knockout mice in Schistosoma japonicum infection and in IgE-FcεRIα-mediated cutaneous anaphylaxis. Thus, we uncovered a novel pathway for the control of FcεRIα expression by its co-expressed natural antisense transcript.

**IMPORTANCE** FcεRIα is responsible for high-affinity binding with the Fc portion of IgE, which is critical for IgE-dependent disease responses such as allergy responses and anti-parasite immunity. FcεRIα is expressed on a few cell types, including mast cells and basophils. Although the expression of FcεRIα is known to be promoted by the IL-3-GATA-2 pathway during its differentiation, the mechanism by which FcεRIα expression is maintained remains unknown. In this study, we discovered that a natural antisense transcript, *FCER1A*-AS, is co-expressed with the sense transcript. The presence of *FCER1A*-AS is essential for sense transcript expression in mast cells and basophils, but not for the differentiation of these cells through *cis*-regulation. Like FcεRIα knockout mice, mice lacking *FCER1A*-AS also exhibit reduced survival after Schistosoma japonicum infection and a lack of IgE-mediated cutaneous anaphylaxis. Thus, a novel pathway for regulating IgE-mediated allergic diseases through noncoding RNAs has been revealed.

## INTRODUCTION

The high-affinity receptor for immunoglobulin E (FcεRI) is known to be critical for IgE-mediated allergic diseases, including asthma, rhinitis, and food allergy, and is probably also critical for certain antibody-mediated autoimmune diseases (1). Moreover, FcεRIα also plays a beneficial role in anti-helminth immunity and its associated immunopathologies (2). Each FcεRI is a tetrameric molecule composed of one α, one β, and two γ chains (αβγ2) (3). The α chain of FcεRI (FcεRIα) confers it with high-affinity binding with the Fc portion of IgE, the β chain amplifies the signal, and the γ chain mediates signal transduction. FcεRIα is almost exclusively expressed on mast cells and basophils in both mice and humans. Recent studies have revealed that FcεRIα may also be expressed on dendritic cells (4, 5) and monocytes (6). Cross-linking of FcεRIα by an immune complex composed of antigens and antigen-specific IgEs is the major mechanism that activates mast cells and basophils, which can play beneficial and pathological roles in helminth infection and allergic diseases. Thus, studying the regulation of FcεRIα expression should have a great impact on improving our understanding of IgE-FcεRIα-mediated diseases.

We previously demonstrated that FcεRIα-positive cells, including dendritic cells, can be found in secondary lymphoid organs in helminth infection. These cells all display a strong capability to induce Th2 cells *in vitro* (5). To explore the roles of FcεRIα-positive cells *in vivo*, an FcεRIα-DTR mouse was constructed by inserting a diphtheria toxin receptor (DTR) into the 3′ UTR (untranslated region) of the *FCER1A* gene locus in the hope that FcεRIα-expressing cells would be specifically abrogated upon diphtheria toxin administration (7). Unexpectedly, correctly gene-edited FcεRIα-DTR mice failed to express FcεRIα mRNA and protein even though the coding sequences were not altered. We pursued and uncovered a novel regulation of FcεRIα by its co-expressed antisense transcripts.

It is currently well known that the expression of many genes is regulated by noncoding RNAs (ncRNAs) via complicated mechanisms. ncRNAs include rRNAs, tRNAs, small nuclear/nucleolar RNAs, microRNAs, and long noncoding RNAs (lncRNAs). lncRNAs may include long intergenic ncRNAs, natural antisense transcripts (NATs), and long intronic ncRNAs; as their name indicates, NATs are transcripts from the strand opposite of the protein-encoding strand (8).

We found that when transcription of the mouse *FCER1A* gene was induced in interleukin (IL)-3-treated bone marrow cells or *FCER1A* was stably transcribed in an FcεRIα-bearing mast cell line, a fully complementary antisense RNA of *FCER1A* (*FCER1A*-AS) was also transcribed in parallel. Further investigations showed that these natural full-length antisense transcripts have a positive effect on sense *FCER1A* expression (*FCER1A*-S). Deficiency of *FCER1A*-AS transcription *in vivo* is always associated with a deficiency of *FCER1A*-S transcription, which results in a lack of IgE-mediated cutaneous allergic reactions and reduced survival following parasite infection.

## RESULTS

### FcεRIα-DTR mice displayed a similar phenotype to FcεRIα-KO mice in *Schistosoma japonicum* infection and IgE-FcεRIα-mediated cutaneous anaphylaxis.

**FIG 1:**
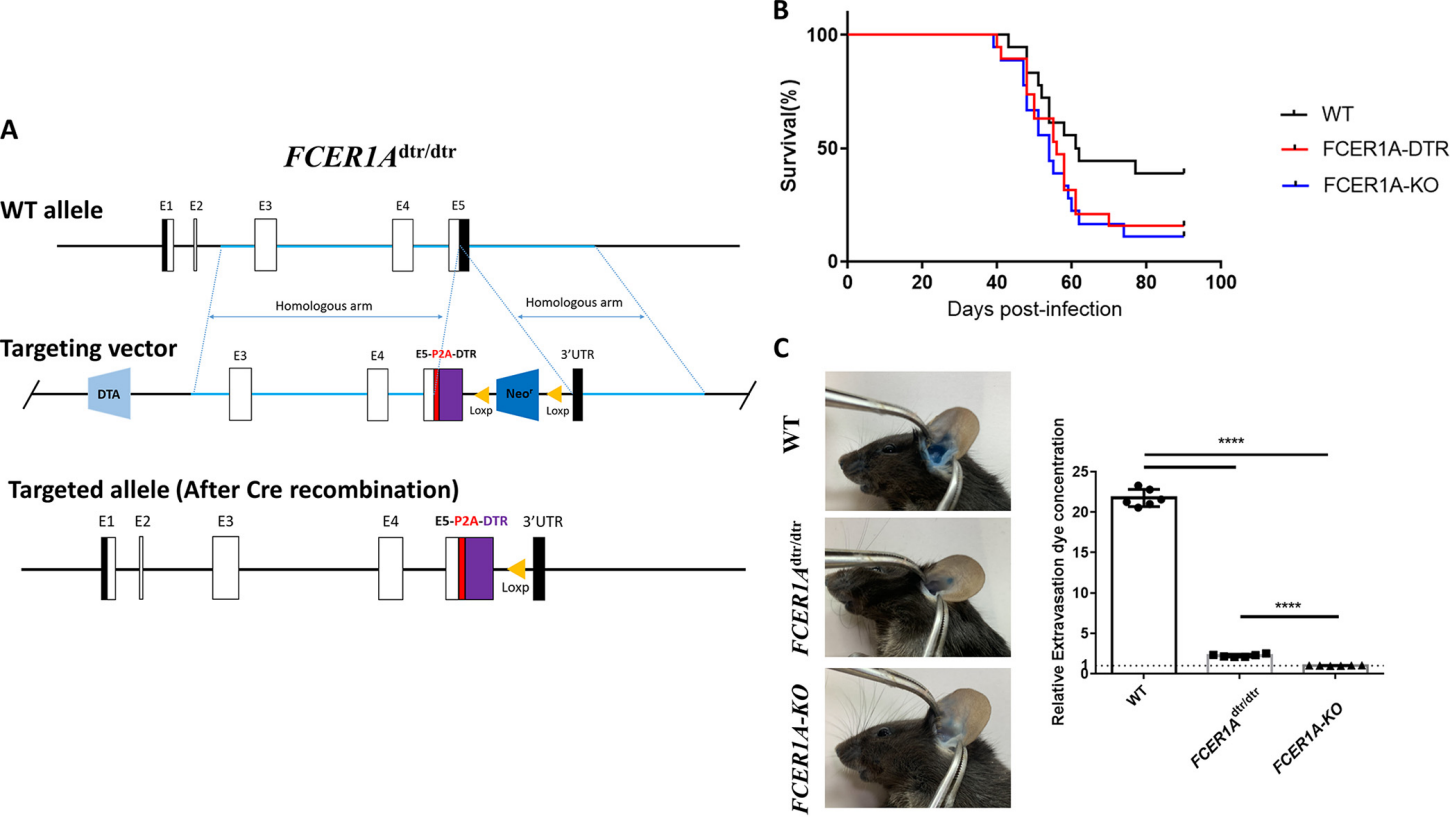
In-frame gene editing in *FCER1A*^dtr/dtr^ mice resulted similar phenotypes in disease models as in FcεRIα deficiency in *FCER1A*-KO (knockout) mice. (A) Gene editing strategy for *FCER1A*^dtr/dtr^. *FCER1A*^dtr/dtr^ mice were generated by introducing a P2A-DTR-*loxP*-Neor-*loxP* cassette into the 3’UTR of FCERIA allele (before stop codon) by homologous recombination. The coding sequence of FcεRIα (α-subunit of the high-affinity receptor for the Fc portion of immunoglobulin E) remained intact. (B) Survival curves of Schistosoma japonicum-infected wild-type (WT), FCER1A^dtr/dtr^, and *FCER1A*-KO mice. Combined data from three independent experiments with WT (*n* = 18); FCER1A^dtr/dtr^ (*n* = 19), and *FCER1A*-KO mice (*n* = 18). (C) Left panel: representative photographs of ear extravasation of dye in WT, *FCER1A*^dtr/dtr^ and *FCER1A*-KO mice during passive cutaneous anaphylaxis. Right panel: quantification of Evan’s blue extravasation in mouse ears following antigen challenge. Relative extravasation dye concentration is the amount of dye obtained in each group versus that in homozygous *FCER1A*-KO mice. Data are means ± standard deviation (SD) from six ears of three mice. ****, *P* < 0.0001.

FcεRIα-DTR mice were constructed by incorporating sequences encoding human DTR and self-cleaving peptide (P2A) before the 3′ UTR without disturbing protein-encoding sequences of FcεRIα; this was designated *FCER1A*^dtr/dtr^ (Fig. 1A). The FcεRIα knockout mice (*FCER1A-*KO) were homozygous mice in which the entire protein-encoding sequence of FcεRIα was replaced with DTR-P2A-GFP (GFP, green fluorescent protein) (Fig. S1A in the supplemental material). Mice of both lines were fertile and healthy. The gene-editing schemes of both lines are illustrated in Fig. 1A and Fig. S1A, and the sequences of the edited region were confirmed in-frame by genomic sequencing.

When wild-type (WT), homozygous *FCER1A*^dtr/dtr^, or *FCER1A-*KO mice were infected with Schistosoma japonicum, the *FCER1A-*KO mice showed a 88% mortality rate, which is significantly higher than the 61% mortality rate in wild-type mice (Fig. 1B). To our surprise, homozygous *FCER1A*^dtr/dtr^ (without DT administration) mice showed a mortality rate of 84%, similar to the *FCER1A-*KO mice, even though the encoding sequences for FcεRIα were not altered during the gene editing process.

We then studied passive cutaneous anaphylaxis reaction (PCA), the pathology of which solely relies on the presence of FcεRIα (9, 10), in wild-type, homozygous *FCER1A*^dtr/dtr^, and *FCER1A-*KO mice. PCA was induced by skin sensitization via subcutaneous injection of anti-DNP (2,4-dinitrophenol) IgE into the ears, followed by intravenous challenge with the antigen DNP. The severity of PCA was reflected by the amount of Evans blue extravasation, which was administered together with the DNP via the bloodstream, in the ears. As expected, *FCER1A-*KO mice displayed 10-fold less extravasation than WT mice as measured by the amount of Evans blue in sensitized ears (Fig. 1C). More importantly, equally diminished extravasation was found in *FCER1A*^dtr/dtr^ homozygous mice.

Insertion of a DTR element into the 3′ UTR of a targeted gene is a common strategy used to achieve ablation of cells expressing the target gene (7). However, the unusual similar phenotype found between FcεRIα-DTR and FcεRIα-KO mice strongly indicated that FcεRIα gene expression was disabled in FcεRIα-DTR mice.

### Deficiency of *FCER1A* expression in *FCER1A*^dtr/dtr^.

**FIG 2:**
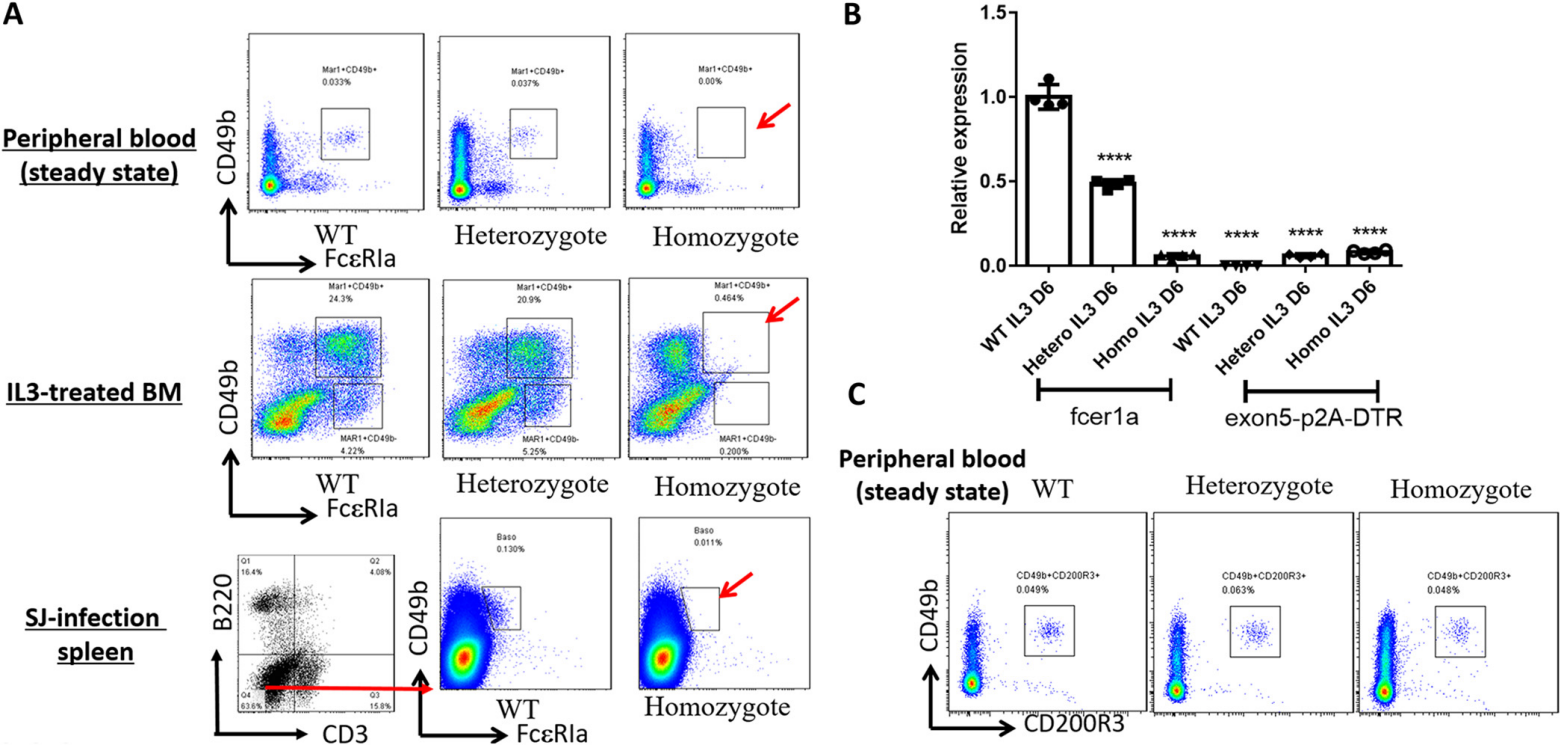
Deficiency of *FCER1A* expression in FCER1A^dtr/dtr^. (A) Fluorescence-activated cell sorter (FACS) analysis of cell surface expression of FcεRIα in normal peripheral blood cells, 6th day interleukin (IL)-3-induced bone marrow cells, and S. japonicum-infected splenocytes in WT mice and heterozygous and homozygous *FCER1A*^dtr/dtr^ mice. In the case of S. japonicum infection, detection of FcεRIα-positivity was gated on Non-B and non-T (NBNT, CD3-B220-) cells. Data are representative of three independent experiments. (B) Quantitative reverse transcription-PCR (RT-PCR) of *FCER1A* or inserted diphtheria toxin receptor (DTR) gene from 6th-day IL-3-treated bone marrow cells in WT and heterozygous or homozygous *FCER1A*^dtr/dtr^ mice. RNA was reverse transcribed by oligo(dT). *FCER1A* expression levels in each setting are shown relative to that in WT mice. Data are means ± SD of four separate animals from each model. ****, *P* < 0.0001. (C) Basophils are not affected in FCER1A^dtr/dtr^. Basophils in peripheral blood were measured by expression of CD49b and CD200R3 in WT and heterozygous or homozygous FCER1A^dtr/dtr^ mice.

When we examined expression of FcεRIα mRNA transcripts or proteins in homozygous *FCER1A*^dtr/dtr^ mice, we found defective FcεRIα expression under various conditions. First, FcεRIα-positive cells detected by fluorescence-labeled anti-FcεRIα monoclonal antibodies (MAR-1) were almost diminished in peripheral blood of homozygous animals, 6th-day IL-3-treated bone marrow cells, and splenocytes from S. japonicum-infected mice in which FcεRIα-positive cells were reported to be elevated (5) (Fig. 2A). Second, transcription of *FCER1A* or the inserted gene DTR, as shown in Fig. 2B, was also undetectable in homozygous *FCER1A*^dtr/dtr^ mice. In both cases, the failure of transcription at the *FCER1A* locus following gene editing was not due to a lack of either FcεRIα-competent cells or IL-3-responsiveness. The percentage of FcεRIα-competent cells such as basophils, as measured by CD200R3 and CD49b in peripheral blood and CD200R3 expression (11) induced by IL-3 in bone marrow cells, were comparable between gene-edited and WT mice (Fig. 2C).

In conclusion, we postulated that an unidentified sequence-associated mechanism may control FcεRIα gene expression. The gene-editing procedure performed in *FCER1A*^dtr/dtr^ mice may have disrupted the integrity of this sequence-associated mechanism; thus, the *FCER1A*^dtr/dtr^ animals displayed a similar phenotype to *FCER1A-*KO mice (Fig. 1).

### Identification of mouse *FCER1A* natural antisense transcript.

In *FCER1A*^dtr/dtr^, DTR is inserted in-frame before the 3′ UTR, and thus the coding sequence for FcεRIα is unaltered. Conceivably, insertion of a gene segment at the 3′ end may result in dysregulated expression of antisense transcripts, if there is any. Furthermore, both stimulatory and suppressive effects on transcription of sense strands by antisense RNA have been reported (12–15).

We first explored whether antisense transcripts were present in FcεRIα-expressing cells. Since FcεRIα expression is largely limited to basophils and mast cells, we used IL-3-treated bone marrow cells to explore whether antisense transcripts were made during transcription of FcεRIα. IL-3 has been shown to induce differentiation of bone marrow cells into FcεRIα-expressing cells, including both basophils and mast cells, potently by itself *in vitro* (5, 16, 17).

**FIG 3:**
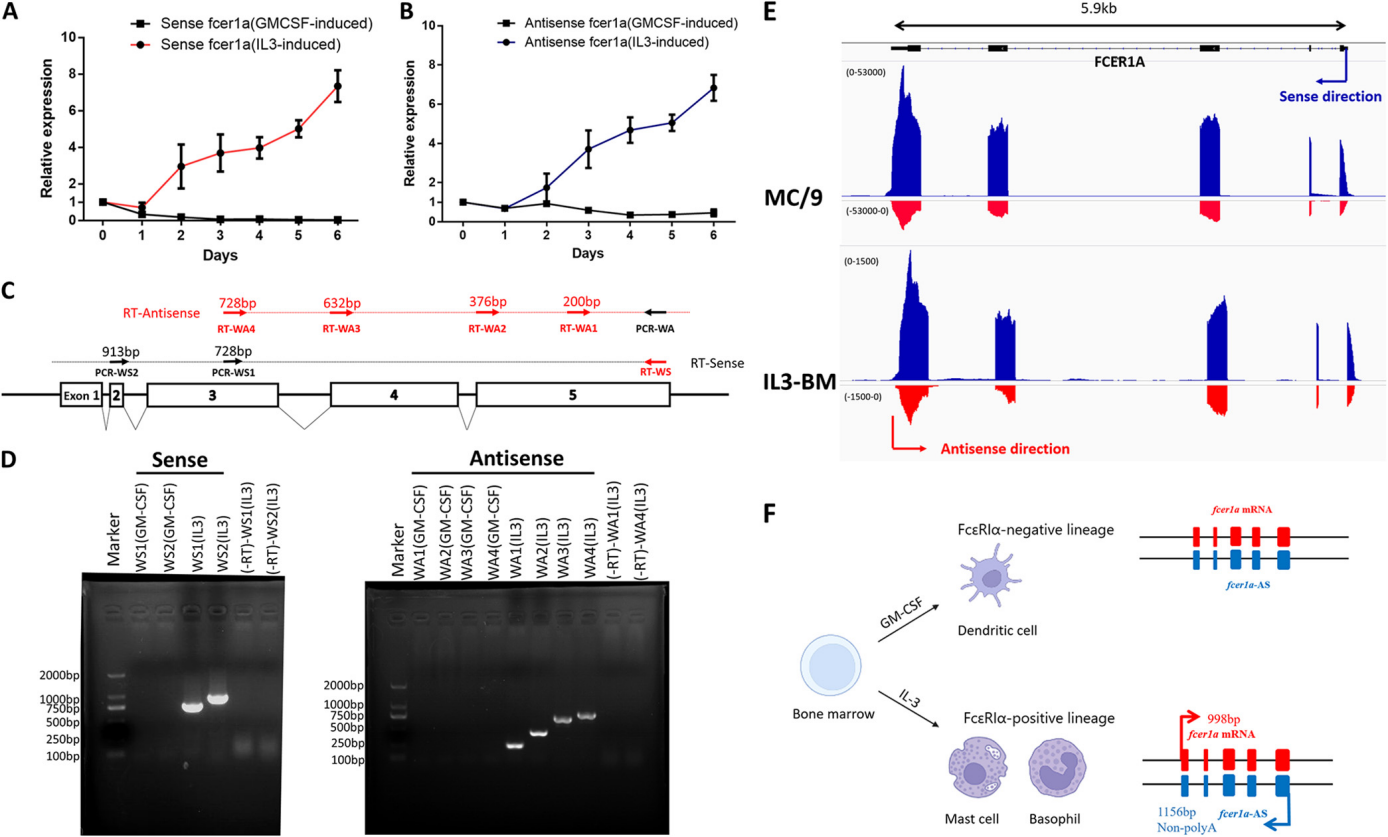
Antisense transcript of *FCER1A* (*FCER1A*-AS) is expressed along with its sense transcript (*FCER1A*-S). (A and B) Time kinetics of sense (A) and antisense (B) of *FCER1A* expression from IL-3 (circle)- or granulocyte-macrophage colony-stimulating factor (GM-CSF, square)-treated bone marrow cells. Combined data show expression level relative to that at time 0 for *n* = 4 independent animals. (C) RT-PCR primer design for detection of sense (black dotted line) and antisense (red dotted line) of *FCER1A*. RT primers (red arrows) for sense (WS) or antisense (WA1 to WA4) and PCR primers (black arrows). (D) Gel analysis of RT-PCR products of sense (left panel) or antisense (right panel) of *FCER1A* from 6th-day IL-3- or GM-CSF-treated bone marrow cells. The “-RT” column shows PCR amplification of DNase-treated RNA without RT for genomic DNA contamination control. Data from one of at least three independent experiments are shown. (E) Strand-specific RNA sequencing (RNA-seq) profiles of *FCER1A* genes from MC/9 and IL-3-treated bone marrow cells. *y* axis reflects counts of RNA-seq reads. Arrows indicate the direction of sense (blue) or antisense (red) transcription. Black line on top indicates known *FCER1A* gene reference. (F) Illustration of *FCER1A*-S and *FCER1A*-AS being co-expressed only in IL-3-induced FcεRIα-positive cells. *FCER1A-*AS is 158 bp longer than its sense counterpart and lacks a poly(A) tail.

The appearances of sense transcripts (*FCER1A*-S) and putative antisense transcripts of *FCER1A* (*FCER1A*-AS) were examined in bone marrow cells treated with IL-3 at various time points. cDNAs for sense and antisense strands were synthesized by strand-specific reverse transcription (RT) using RT primers specific for sense strand (RT-WS) or putative antisense strand (RT-WA). Quantitative PCR amplification of strand-specific cDNAs was then performed using primers (Fig. S2A, Table S1) designed between exons 3 and 4 with a product about 109 bp in size. As shown in Fig. 3A, *FCER1A* sense transcripts (*FCER1A*-S) were almost undetectable in untreated bone marrow cells by quantitative RT-PCR. Treatment with granulocyte-macrophage colony-stimulating factor (GM-CSF) failed to induce any significantly increased expression of *FCER1A*-S and was thus used as a negative control for cytokine treatment. Following IL-3 treatment, *FCER1A*-S became detectable 2 days after treatment. *FCER1A*-S levels were significantly increased 6 days after IL-3 treatment compared to those in untreated or GM-CSF-treated cells (Fig. 3A). Notably, *FCER1A*-AS displayed similar time kinetics to *FCER1A*-S following IL-3 treatment. It was not detectable in freshly isolated bone marrow cells or GM-CSF-treated cultures and was also highly induced 6 days after IL-3 treatment (Fig. 3B).

To further explore whether antisense transcripts also overlapped other exons and UTRs of *FCER1A*, we performed antisense strand-specific reverse transcription reactions using primers specific for the 5′ UTR (RT-WA1), exon 5 (RT-WA2), exon 4 (RT-WA3), and exon 3 (RT-WA4), as shown in Fig. 3C. As shown in Fig. 3D, similar to the induction of sense transcripts by IL-3 treatment, all four antisense transcripts detected by the corresponding primers were clearly induced as well. The presence of *FCER1A*-AS was also verified in MC/9 cells (data not shown), a mouse mast cell line which expresses high levels of *FCER1A*-S (11). Lastly, the presence of *FCER1A*-AS was demonstrated by strand-specific RNA sequencing (RNA-seq) and bioinformatics analysis in both IL-3 treated bone marrow cells and MC/9 cells (Fig. 3E). Remarkably, RNA-seq also revealed that *FCER1A*-AS fully overlaps transcribed regions, but not intron regions. Therefore, when the *FCER1A* sense strand is transcribed, a full-length antisense transcript is created in parallel.

Rapid amplification of cDNA ends (RACE) was performed on RNA isolated from MC/9 cells or 6th-day IL-3-treated bone marrow cells to identify the starting and ending sites of *FCER1A*-AS transcription. The results demonstrated that *FCER1A*-AS is 158 bp longer than its sense counterpart and lacks a poly(A) tail (Fig. S2B). Sanger sequencing of the PCR products of full-length *FCER1A*-AS verified that *FCER1A*-AS is fully complementary to all transcribed regions, including all five exons and the 5′ and 3′ UTRs, but not to intron regions (Fig. S2C). There is no coding capacity predicted for *FCER1A*-AS *in silico* (Fig. S2E, http://cpc2.gao-lab.org/) (18). When the RNA stability of both sense and antisense strands was examined in MC/9 cells, the half-life for *FCER1A*-AS was approximately 13 h, nearly 3 h longer than that of *FCER1A*-S (Fig. S2D).

These data firmly established that natural antisense transcript of FcεRIα (*FCER1A*-AS) is co-expressed with the cognate sense transcript (*FCER1A*-S) when *FCER1A*-S is either steadily expressed or induced by IL-3 (Fig. 3F).

### Knockdown of *FCER1A*-AS reduces FcεRIα mRNA and protein expression.

**FIG 4:**
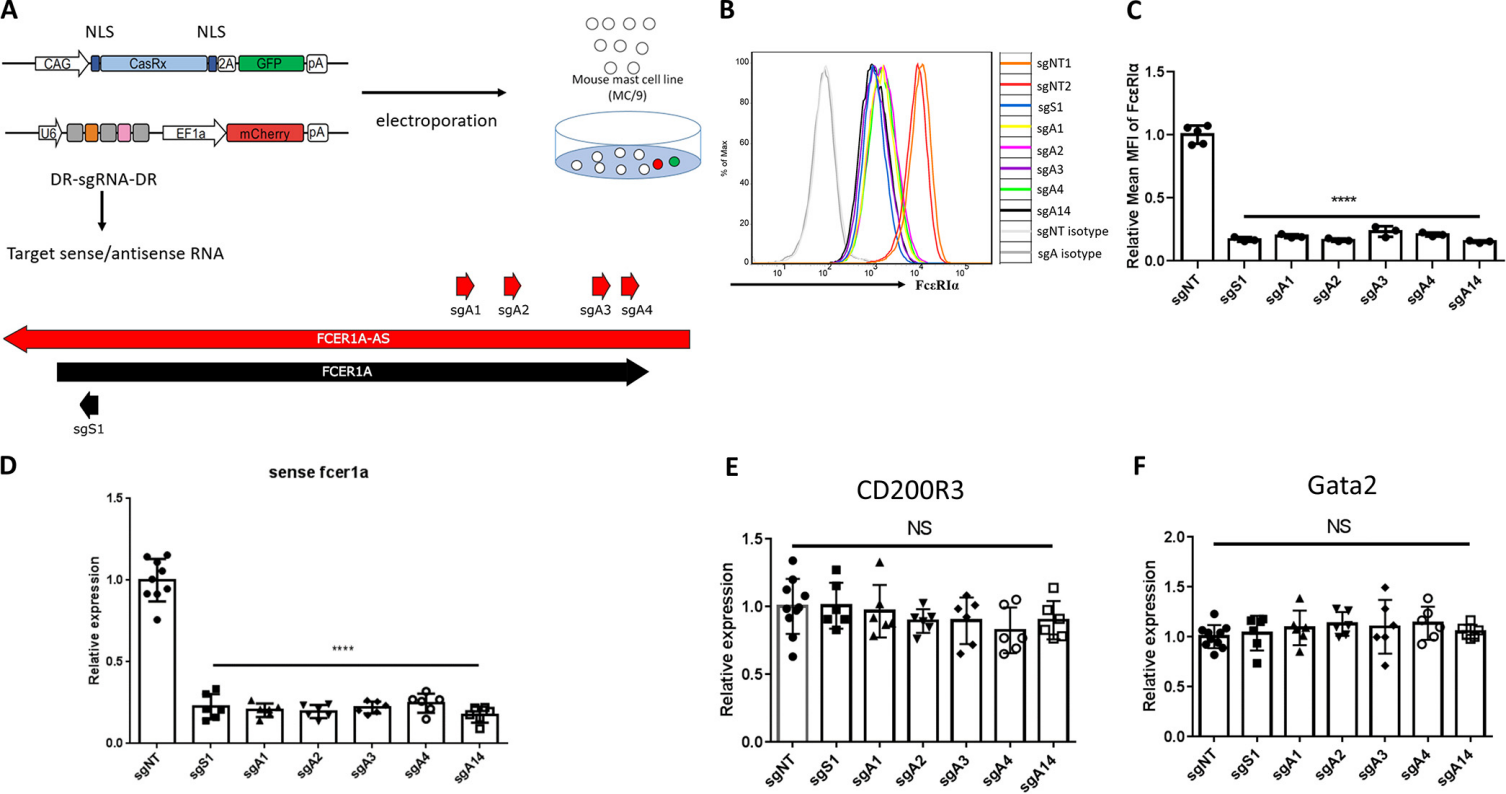
CasRx-mediated knockdown of *FCER1A-*AS in MC/9 resulted in defective expression of *FCER1A-*S. (A) Strategies for CasRx-mediated knockdown of *FCER1A*-AS or *FCER1A*-S in MC/9. Four differently targeted guide RNAs (sgRNAs) (A1, A2, A3, and A4) and one multiplexed sgRNA combining all four sgRNAs (A1 to -4) were designed to knock down *FCER1A*-AS, and one sgRNA (S1) was designed to knock down *FCER1A*-S. MC/9 cells were co-transfected by electroporation with expression plasmids for sgRNA and CasRx. (B) FACS analysis of surface expression of FcεRIα following *FCER1A*-AS-sgRNA (sgA) or FCER1A-S-sgRNAs (sgS) transfection. FACS staining of co-transfected cells gated on cells positive both for green fluorescent protein (GFP+) and mCherry (mCherry+) is shown. Non-targeting sgRNAs (sgNT) and isotype staining (gray) were used as controls. Histogram shown is representative of three independent experiments. (C) Summarized mean fluorescence intensity (MFI) of FACS staining of FcεRIα relative to the MFI of sgNT. *n* = 3 independent experiments. (D) Strand-specific quantitative RT-PCR of *FCER1A*-S expression following *FCER1A*-AS-sgRNA (sgA) or FCER1A-S-sgRNAs (sgS) transfection. Expression is shown as the reduced levels in each sgRNA knockdown treatment detected by qRT-PCR relative to the control sgNT. Data are means ± SD of six different experiments. (E and F) Quantitative RT-PCR of *GATA2* and *CD200R3* in MC/9 cells following *FCER1A*-AS-sgRNA (sgA) or *FCER1A*-S-sgRNAs (sgS) transfection. RNA from each group was reverse transcribed by random hexamers. Expression levels are shown relative to that of the control sgNT. *n* = 6 different experiments. ****, *P* < 0.0001; NS, no significant difference.

To explore whether *FCER1A*-AS played any regulatory role in *FCER1A*-S expression, we performed RNA knockdown of *FCER1A*-AS in MC/9 cells. The recently developed CRISPR/RfxCas13d (CasRx) technique is used to selectively knock down the antisense strand, a technique based on the unique RNA-guided RNA-interfering activity possessed by Cas13 (19–21). We designed our different guide RNAs (gRNAs) targeting the 5′ regions of *FCER1A*-AS (*FCER1A*-AS-gRNAs) (Fig. 4A). Two non-targeting gRNAs (NT-gRNAs) that do not target *FCER1A*-AS or any known sequences in mouse genomes were used as controls (Fig. 4A).

Plasmids expressing gRNAs or CasRx were co-transfected into MC/9 cells by electroporation. Transfection of *FCER1A*-AS-gRNAs or NT-gRNAs slightly reduced cell viability (Fig. S3A). Significant knockdown of *FCER1A*-AS by all four *FCER1A*-AS-gRNAs, but not by the NT-gRNAs, was observed 48 h after co-transfection (Fig. S3B).

Significantly, these cells also displayed reduced FcεRIα expression. The percentage of FcεRIα-expressing MC/9 cells (Fig. 4B and Fig. S3C), levels of FcεRIα expression on cell surfaces as measured by mean fluorescence intensity (Fig. 4C), and levels of FcεRIα mRNA transcription were all markedly decreased as a result of *FCER1A-AS* knockdown (Fig. 4D). No inhibition of sense transcript expression was found when NT-gRNAs/CasRx was transfected. No further inhibition was seen when multiple sites on *FCER1A*-AS were targeted by multiple gRNA strings. Importantly, knockdown of *FCER1A*-AS did not result in alterations of other known mast cell-associated marker molecules examined, including GATA-2 and CD200R3 (Fig. 4F and E). Therefore, loss of *FCER1A*-AS caused a loss of *FCER1A*-S. *FCER1A*-AS plays a critical positive role in regulating the transcription of its co-expressed sense strand.

### Deficiency of *FCER1A*-S was associated with deficiency of *FCER1A*-AS in *FCER1A*^dtr/dtr^ mice.

**FIG 5:**
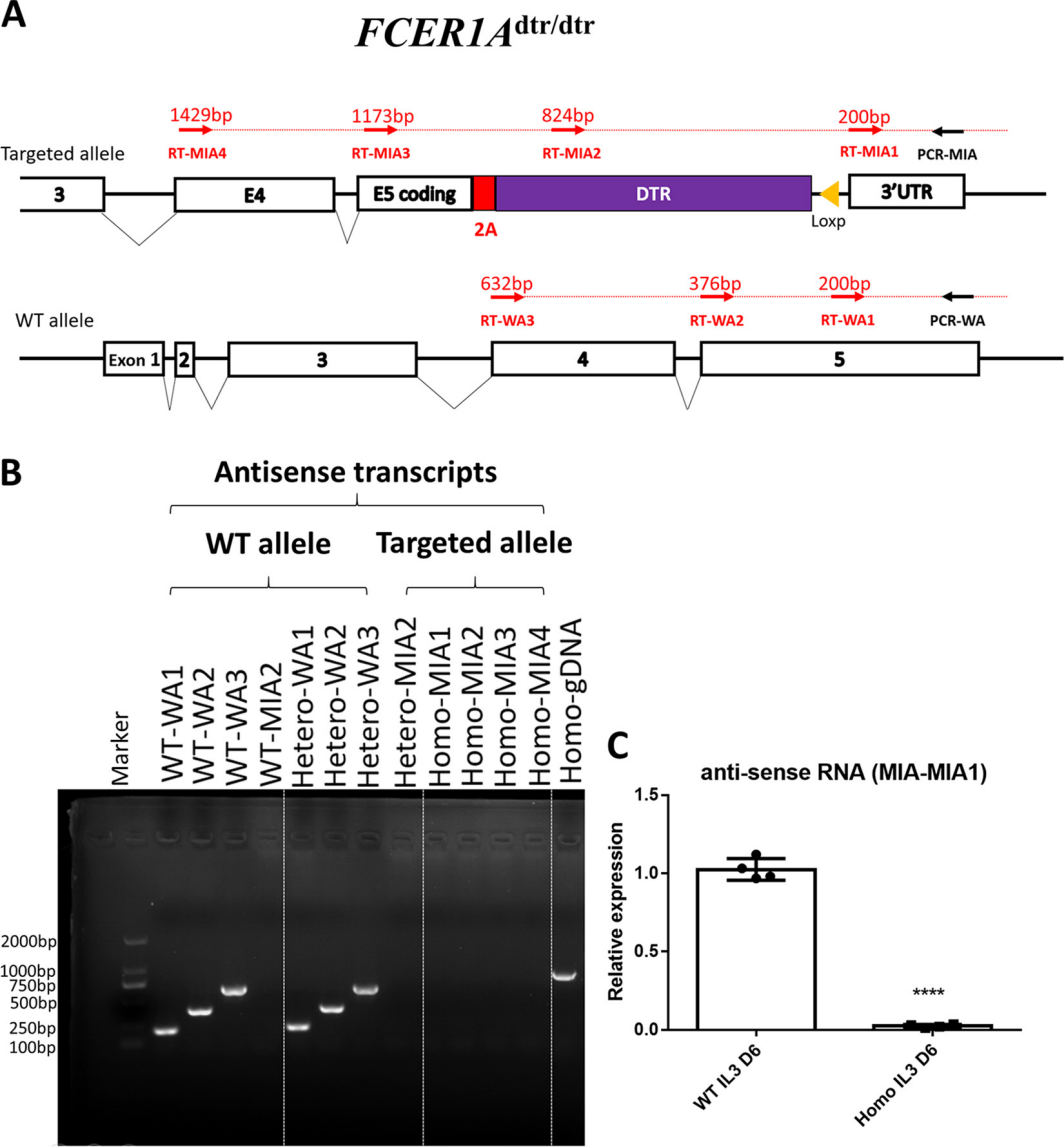
Absence of *FCER1A*-AS is correlated with loss of *FCER1A*-S expression in *FCER1A*^dtr/dtr^. (A) RT-PCR primer design for detection of antisense transcripts at *FCER1A* loci in WT mice and heterozygous and homozygous *FCER1A*^dtr/dtr^ mice. RT primers (red arrows) in WT (WA1 to WA4) and *FCER1A*^dtr/dtr^ mice (MIA1 to MIA4). Common PCR primer (black arrows) is used together with respective RT primers to amplify PCR products. (B) RT-PCR analysis of antisense transcripts of *FCER1A* loci from 6th-day IL-3-treated bone marrow cells in WT and *FCER1A*^dtr/dtr^ mice. PCR of genomic DNA (gDNA) from homozygous mice was included as a positive control for PCR. Data are representative of three independent experiments. (C) Strand-specific quantitative RT-PCR analysis of *FCER1A*-AS from *FCER1A*^dtr/dtr^. RNA from each group was reverse transcribed by antisense strand-specific primer (RT-WA1 for WT; RT-MIA1 for *FCER1A*^dtr/dtr^), and the 3′ UTR was selected for final qPCR detection. Relative expression indicates the ratio relative to the WT. Data are the means ± SD of four separate animals from each model. ****, *P* < 0.0001.

Because we had just determined that antisense transcript *FCER1A-*AS is required for expression of its co-expressed sense partner *FCER1A-*S in MC/9 cells, we then examined the expression of antisense transcripts in 6th-day IL-3 treated bone marrow cultures from *FCER1A*^dtr/dtr^ mice. Primers for strand-specific reverse transcription of targeted alleles were designed (Fig. 5A). As shown in Fig. 5B and C, homozygous *FCER1A*^dtr/dtr^ mice exhibited a deficiency in antisense transcripts of the targeted alleles, i.e., *FCER1A*-AS or antisense of the DTR gene in *FCER1A*^dtr/dtr^. Since the expression of FcεRIα sense transcripts was deficient in homozygous *FCER1A*^dtr/dtr^ mice, we believe that the co-expression pattern found on S/AS of *FCER1A* is critical for successful FcεRIα expression *in vivo*.

Notably, although the sense transcript was undetectable in IL-3-treated bone marrow cells of homozygous *FCER1A*^dtr/dtr^ mice (Fig. 2B), *FCER1A*-S was readily detectable but at reduced levels in heterozygous *FCER1A*^dtr/dtr^ mice compared to wild-type mice (Fig. 2B). In parallel, the antisense transcript *FCER1A*-AS was also detectable in heterozygous *FCER1A*^dtr/dtr^ mice (Fig. 5G). This strongly suggests that the presence of *FCER1A*-AS from unedited chromosome cannot rescue the inhibited transcription of sense transcripts from the edited chromosome. Therefore, the positive regulation of sense strand transcription by its cognate transcribed antisense strand on *FCER1A* appears to occur in a highly spatially-restricted, *cis*-acting manner.

### Analysis for initiation potentials of *FACERI*-AS transcription.

**FIG 6:**
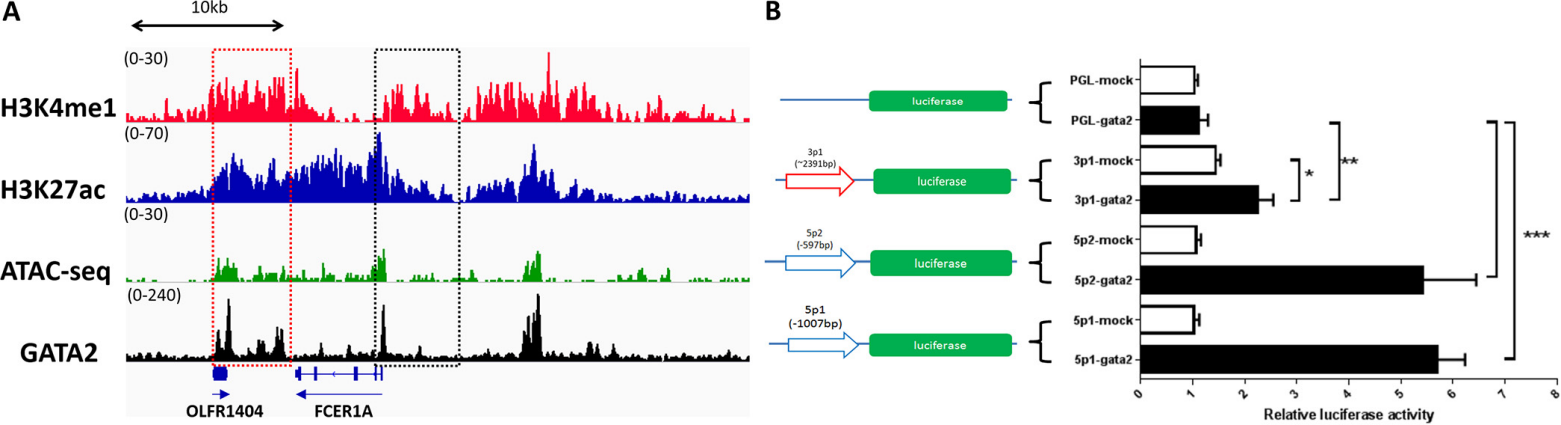
Putative transcription initiation analysis for *FACERIA*-AS. (A) Representative tracks of H3k4me1/H3k27ac/GATA-2 by ChIP-seq (chromatin immunoprecipitation sequencing) and ATAC-seq (assay for transposase-accessible chromatin with high-throughput sequencing) for the *FCER1A* locus in bone marrow-derived mast cells. Data are shown as read density around the *FCER1A* locus. IGV tracks are generated from one biological sample, representing two biological replicates with similar patterns. Raw data were retrieved from GEO under accession no. GSE145542 and GSE145544. (B) GATA-2 transactivates *FCER1A*-AS putative promoter at a lower level than *FCER1A*-S. Reporter plasmids carrying promoter regions of *FCER1A*-S (−1,007/+1,5p1; −597/+1,5p2), *FCER1A*-AS (−2,391/+1,3p1), or control plasmid pGL-4 were transfected into HEK293T cells. GATA-2 expression plasmid pCDNA3.1 (+)-GATA2 (GATA2) and pCDNA3.1 (+) (mock) were co-transfected. Promoter activities were determined by comparing luciferase activity between GATA-2 and mock. Data are means ± SD of four independent experiments.

To identify the potential mechanisms involved in allowing the transcription of antisense *FCER1A*, we analyzed the gene configuration of the *FCER1A* locus in bone marrow-derived FcεRIα-expressing mast cells using NCBI Gene Expression Omnibus (GEO) data sets (accession no. GSE145542 and GSE145544) (22). We found that the binding signals for H3K4me3 and H3K27ac, two types of histone modifications with upregulating activities on gene transcription, are enriched not only at the 5′-end promoter regions of *FCER1A-*S, as expected, but also with comparable peak values at the putative 5′-end promoter flanking region of *FCER1A-*AS in Bone marrow-derived mast cells (BMMC) (Fig. 6A). A similar signal pattern was also found using ATAC-Seq (assay for transposase-accessible chromatin with high-throughput sequencing) and ChIP-Seq (chromatin immunoprecipitation sequencing) regarding the binding activity of GATA-2, the key positive transcriptional factor for *FCER1A-*S transcription during differentiation of FcεRIα-expressing cells (16, 23). Because the nearest downstream gene, OLFR1404, was not expressed in mast cells (Fig. S4B), we believed that the gene configuration upstream of *FCER1A-*AS favors its transcription.

We then examined the potential role of GATA-2 in the transactivation of antisense promoter using a classic luciferase reporter assay. Compared to the significant GATA-2-associated promoter activity on sense direction, we found weaker GATA-2-associated promoter activity for *FCER1A*-AS transcription 2.4 kb downstream from the 3′ UTR of the FcεRiα gene, i.e., 2.4 kb upstream from the reverse strand (Fig. 6B). Therefore, GATA-2 may contribute to antisense strand transcription with weakened strength.

## DISCUSSION

In this study, we found that the presence of FcεRIα prolongs survival in mice infected with S. japonicum. More significantly, the expression of FcεRIα relies on its co-expressed antisense partner, *FCER1A*-AS. Mice lacking antisense transcripts, as in *FCER1A*^dtr/dtr^, also lack FcεRIα expression, and succumbed more quickly to S. japonicum infection.

FcεRIα produces beneficial effects in defense and immunopathology against several types of parasite infections, including Schistosoma mansoni (24) and Haemaphysalis longicornis ticks (25, 26), etc. Lack of FcεRIα causes increased skin penetration by ticks and worsened liver pathologies in animals infected with S. mansoni. In this study, lack of FcεRIα resulted in reduced survival of infected animals. Infection with helminths such as *Schistosoma* tends to induce Th2-dominated type 2 immunity (5, 27). Furthermore, the level of Th2 response induced can determine survival and immunopathology (28). When Th2 responses were measured in splenocytes infected with S. japonicum, no differences were found between WT and FcεRIα-deficient mice (Fig. S1D and E). This is similar to Th2 responses in S. mansoni infection (24). The mechanisms underlying the survival advantages provided by FcεRIα await further exploration.

Antisense transcripts or natural antisense transcripts are transcripts which are co-expressed with their sense counterparts without encoding detectable proteins or peptides. S/AS co-expression, no longer restricted to imprinted loci seen in chromosome inactivation, is a phylogenetically conserved phenomenon (12). As suggested by large-scale RNA-seq and high-speed computational analyses, it is estimated that 26% of protein-encoding sequences possess overlapping NATs which probably represent 29% of lncRNAs (>200 nucleotides in length) in humans (29, 30). However, these data fail to reveal the presence of NATs in inducible genes. For example, *FCER1A*-AS, described in this study, is not found in several published databases, probably because the expression of *FCER1A*-S is induced. Using IL-3 induction, we were able to identify an almost identical co-expression pattern between *FCER1A*-AS lncRNA and *FCER1A*-S.

We found that *FCER1A*-AS fully overlaps with the sense transcript. The mechanisms for side-by-side expression of S/AS are unknown and it is unclear whether they depend on identical splicing machinery. It seems that the putative promoter region of the antisense direction of *FCER1A* is in a relaxed state, similar to that of the sense direction in FcεRIα-competent cells. GATA-2 only exhibits weak promoting activities, indicating that another initiating force of antisense transcription should exist. Bi-directional promoters, probably rich in CpG islands, may allow cognate NATs to be produced with a so-called divergent head-to-head configuration (31). It is difficult to imagine that this can result in the generation of fully overlapped NATs. The other proposed model is that mobile genetic elements, especially ancient transposon sequences such as MIR and L2, which may be present upstream of antisense sequences, function as promoters to trigger AS transcription when the chromosome structure of the locus relaxes (32, 33). A sequence blast analysis indicated that transposon MIR element is spread within this region (Fig. S4A). Additionally, the transcription of *FCER1A*-AS is due to extended transcription of the neighboring gene located downstream from *FCER1A*. This possibility is excluded because RNA-seq data from FcεRIα-expressing cells failed to collect any signals from the closest downstream neighbor gene, OLFR1404 (Fig. S5B).

It is intriguing that reverse-strand expression is defective in *FCER1A*^dtr/dtr^ mice, suggesting that some unknown sequence-intrinsic mechanism possessed by the *FCER1A* locus may dictate whether or not antisense transcripts can be expressed. Conceivably, these unknown sequence-intrinsic mechanisms could become uncertainties during gene manipulation. For examples, failed expression of mouse siglec H was reported when DTR was inserted before the 3′ UTR of this gene (34). Insertion of a human dihydrofolate reductase resistance cassette downstream from the 3′ UTR of the sexual commitment gene GDV1 in a malaria parasite disrupted the expression of GDV1 antisense RNA, resulting in dysregulated expression of sense GDV1 (35).

Based on the few reported examples of transcriptional interference of sense transcript by AS, AS can only regulate the expression of sense cognate on the same chromosome, so-called *cis*-regulation (36). In the *FCER1A*^dtr/dtr^ mice in this study, loss of *FCER1A*-S and *FCER1A*-AS transcription was found in homozygous mice, whereas heterozygous mice still possessed S/AS transcript expression at reduced levels (Fig. 2A and B and Fig. 5B). These, therefore, provide another strong paradigm for regulation of S/AS in a *cis*-acting manner which only allows gene expression to be affected monoallelically. Epigenetic-like mechanisms have been proposed in understanding the *cis*-regulation of sense transcription by antisense transcripts (33).

Both up- and downregulation are seen in terms of the regulation of sense gene expression by its NATs. The regulation of X chromosome inactivation by XIST/TSIX offered the earliest and best-studied example of repression of gene activity by NATs (12). However, we found a de-repressing effect of *FCER1A*-AS on sense gene expression because the presence of AS is required for the induction of its sense partner. Single-stranded RNA knockdown of *FCER1A*-AS via CasRx resulted in reduction of *FCER1A* mRNA and protein. Similar positive regulatory effects have been reported for the tumor suppressor gene PDCD4, which controls breast cancer progression, and the association of *BACE1* with Alzheimer’s disease pathophysiology, where the stability of mRNAs was found to be promoted by their NATs (13, 14). In this study, we speculated that *FCER1A*-AS may promote *FCER1A* mRNA expression by enhancing transcription initiation.

*FCER1A* is a peculiar gene since its expression is not essential for survival and fertilization (9) and its expression is limited to basophils and mast cells in steady state. Its existence seems to mainly trigger IgE-FcεRIα-dependent reactions to expel unpleasant allergens and worms. Dysregulation of IgE-FcεRIα-dependent reactions can be detrimental. The fine balance between good and ill outcomes of this reaction demands stringent molecular control of FcεRIα expression. FcεRIα expression can be upregulated by cytokines, such as IL-3 and TSLP or the ligand molecule IgE (37–39). In addition to well-known *trans*-regulatory molecules such as GATA-2 and STAT-5 (16, 23), which control FcεRIα expression bi-allelically, our findings demonstrate another layer of monoallelic control of FcεRIα expression. The highly spatially controlled regulation of FcεRIα expression by its co-expressed partner *FCER1A*-AS may suggest another pathway to control IgE-FcεRIα-mediated diseases.

## MATERIALS AND METHODS

### Mice.

Wild-type C57BL/6 mice were purchased from SLAC Laboratory (Shanghai, China). All mice were housed and bred under specific pathogen-free conditions in the Animal Center of Tongji University and used at 6 to 8 weeks of age. All procedures performed on animals in this study were approved by the Committee on the Ethics of Animal Experiments of Tongji University (permit no. TJAA07920101).

*FCER1A*^dtr/dtr^ mice were generated in a B6 background by Cyagen (Suzhou, China) using a homologous recombination strategy as shown in Fig. 1A. A linearized targeting vector containing a P2A-DTR-*loxP*-Neo^r^-*loxP* cassette was delivered to ES cells (C57BL/6) via electroporation. A DTR-containing cassette was placed into the *FCER1A* allele by homologous recombination with homology arms flanking 4.8 kb upstream from exon 3 and 2.8 kb downstream from exon 5, respectively, allowing P2A-DTR to be inserted in-frame before a stop codon. The primers for genotyping are listed in Table S1.

*FCER1A* knockout mice were generated in a B6 background by Biocytogen (Beijing, China) using the CRISPR/Cas9 strategy shown in Fig. S1A. The targeting construct, assembled by PCR and cloning, consists of two homology arms and the DTR-P2A-EGFP sequence, with the 5′ and 3′ UTR sequences remaining intact. One arm is a 1.5-kb fragment spanning upstream from exon 1 up to the start codon containing the entire 5′ UTR; the other arm is a 1.5-kb fragment downstream of the stop codon containing the entire 3′ UTR. Two guide RNAs (sgRNAs) were designed to target regions proximal to the upper and lower homology arms, respectively. SgRNAs and Cas9-expressing vectors along with the targeting constructs were delivered to fertilized zygotes through microinjection. The primers for genotyping are listed in Table S1. Mice of both lines were fertile and healthy.

### Infection.

Gender- and age-matched mice were infected percutaneously with 30 cercariae of S. japonicum, shed from infected Oncomelania hupensis snails provided by the National Institute of Parasitic Diseases in Shanghai, China.

### Cell culturing.

Bone marrow cells were flushed from the femurs and tibiae of 6- to 10-week-old wild-type, *FCER1A*^dtr/dtr^, and *FCER1A*-KO mice. After lysis of red blood cells, isolated cells were cultured in RPMI 1640 medium (Gibco, Invitrogen) containing 10% heat-inactivated fetal bovine serum (FBS; Gibco, Invitrogen) and 50 μM 2-mercaptoethanol (Gibco) at 1 × 10^6^/mL. Next, either 10 ng/mL IL-3 (Peprotech) was added for induction of FcεRIα-expressing cells or 10 ng/mL GM-CSF (Sino Biological) was added for no induction of FcεRIα-expressing cells, with the medium replaced every 2 days. Cells were usually harvested 6 days after treatment.

The mast cell line MC/9 was purchased from ATCC (CRL-8306). MC/9 cells were cultured in Dulbecco’s modified Eagle’s medium (Gibco, Invitrogen, Billings, MT) adjusted to contain 4 mM l-glutamine, 4.5 g/L glucose, and 1.5 g/L sodium bicarbonate and supplemented with 2 mM l-glutamax (Gibco, Invitrogen), 50 μM 2-mercaptoethanol, 10% concanavalin A (Sigma-Aldrich)-stimulated splenocyte supernatant, 10 ng/mL IL-3, and 10% FBS. Medium was replaced every 2 days and cell density was maintained at 1 × 10^6^ cells/mL.

### Generation of plasmids.

The vectors for CasRx and sgRNA expression were kindly provided by Hui Yang (Institute of Neuroscience, Chinese Academy of Sciences, Shanghai, China). To construct sgRNA-expressing vectors, backbone vectors of U6-DR-sgRNA-DR-EF1a-mCherry-SV40 were first digested with NdeI and AsiSI restriction enzymes (New England Biolabs [NEB], Ipswich, MA), followed by ligation with inserted sequences containing various sgRNAs (Table S2), which were synthesized by Sangon Biotech Company (Shanghai, China). MC/9 mast cells were co-transfected by electroporation with 7 μg of CasRx-expressing vector and 11 μg of sgRNA-expressing vector. Next, 5 × 10^6^ MC/9 cells were suspended in 0.5 mL Opti-MEM (Gibco) and kept at room temperature for 10 min before electroporation.

Putative promoter regions for mouse *FCER1A*-S or mouse *FCER1A*-AS were synthesized by Sangon Biotech Company (Shanghai) and inserted into the multicloning sites of pGL-4.17 Basic (Promega, Madison, WI) to generate reporter plasmids for detection of putative promoter activities. GATA-2-expressing vector was constructed by cloning synthesized mouse GATA-2 sequences into eukaryotic expression vector pcDNA3.1 (+) (Invitrogen) with restriction enzymes of NheI and EcoRI (NEB). HEK293T cells were transfected with 3 μg pGL-4.17 reporter plasmid, 3 μg GATA-2 expression plasmid, and 375 ng hRluc (Promega) by Lipofectamine 3000 (Thermo Fisher, Waltham, MA). Luciferase activity was determined using a dual-luciferase assay kit (Promega) as described previously (40).

### Transfection of cells.

Here, 5 × 10^6^ MC/9 cells were suspended in 0.5 mL Opti-MEM (Gibco) and kept at room temperature for 10 min. Cells were then electroporated using Gene Pulser II (Bio-Rad) with pulse conditions of 800 μF and 280 V. Cells were kept at room temperature for additional 10 min before being placed back into a 37°C incubator in MC/9 culturing medium. All detection was performed 48 h after transfection.

### Strand-specific reverse transcription and quantitative real-time PCR.

Total RNA was prepared using RNAiso Plus (TaKaRa Bio, Shiga, Japan). One μg of total RNA was used to synthesize cDNA using a PrimeScript RT reagent kit (RR047A, TaKaRa Bio) according to the manufacturer’s instructions. Briefly, RNA was first treated with gDNA Eraser (TaKaRa Bio) to remove genomic DNA. Next, 1 μg of total RNA was reverse-transcribed in 20-μL volumes for 15 or 20 min with either random hexamers or strand-specific primers of *FCREIA* (Table S1). Then, 1/5 cDNA was subsequently amplified by PCRs at 35 cycles at various annealing temperatures. PCR products were visualized on agarose gels by GoldView. Quantitative real-time PCR was performed using SYBR Premix EX *Taq* (Tli RNaseH Plus; TaKaRa Bio) in a QuantStudio3 thermal cycler (Applied Biosystems, Foster City, CA). All quantitative RT-PCR values for GATA-2 and CD200R3 were first normalized by GAPDH (glyceraldehyde-3-phosphate dehydrogenase). The specific primers used are shown in Table S1.

### Strand-specific RNA sequencing.

RNA from either IL-3-induced bone marrow cells or MC/9 cells was isolated as previously described. Here, 5 μg RNA was used as input material for strand-specific RNA-seq. rRNA was removed by a Epicentre Ribo-Zero rRNA Removal kit (Epicentre), and rRNA-free RNA was obtained by ethanol precipitation. Sequencing libraries were subsequently generated using the NEBNext Ultra Directional RNA Library Prep kit for Illumina (NEB). Briefly, after RNA fragmentation, rRNA-free RNA was subjected to first-strand cDNA synthesis using random hexamer primers and M-MuLV Reverse Transcriptase. Second-strand cDNA synthesis was performed using DNA polymerase I and RNase H with dTTP being replaced by dUTP. After end repair, poly(A) tailing, adaptor sequence addition, and U excision, the strand-specific libraries were sequenced by Novogene Bioinformatics Institute (Beijing, China) on an Illumina HiSeq 4000 platform. Finally, 12G of 150-bp paired-end reads were generated.

### RNA-seq data analysis.

Sequence data from MC/9 libraries was aligned to mouse genome build mm10 using Hisat2 software version 2.2.1 (41, 42). SAMtools version 1.14 (http://www.htslib.org/) was used to detect and analyze regions of signal on each strand of *FCERIA* location. Bam2wig.py converts all types of RNA-seq data from BAM format into wiggle format. Data visualization was aided by Integrated Genomics Viewer (IGV) software based on read depth information.

### Rapid amplification of cDNA ends.

5′ and 3′ RACE was performed with a SMARTer RACE 5′/3′ kit (TaKaRa Bio) following the manufacturer’s instructions. In brief, total RNA was extracted from IL-3-induced bone marrow cells or MC/9 cells. First-strand cDNA was synthesized with gene specific primers as shown in Table S1. Touchdown PCR was then performed to amplify different transcripts. Poly(A) tails were added to RNA (TaKaRa Bio) following the manufacturer’s instructions. PCR products were gel-extracted for Sanger sequencing.

### Flow cytometry analysis of surface molecules.

Fluorescence-activated cell sorter (FACS) staining of surface molecules and intracellular cytokines on cultured cells or splenocytes was performed as described previously (5). For peripheral blood cells, orbit blood was collected from isoflurane-anesthetized mice. Blood-collecting tubes were coated with 0.1 M EDTA to prevent clotting. The blood samples were then subjected to fluorescence-labeled antibody staining before red blood cells were dissolved by a lysing solution (LSB01, MultiSciences Biotech, Hangzhou, China). Fluorescence-labeled antibodies and reagents for live cell detection are all listed in Table S3. Final fluorescence detection was performed using FACSVerse (BD Biosciences, San Jose, CA) or CytoFLEX LX (Beckman Coulter, Pasadena, CA). Flow cytometry data analysis was analyzed by FlowJo software (FlowJo LLC) or CytExpert (Beckman Coulter).

### Passive cutaneous anaphylaxis.

PCA was performed as previously described (9, 10). Animals were primed intradermally in both sides of the ear pinna with 100 μg murine anti-DNP IgE (Sigma-Aldrich) dissolved in 20 μL phosphate-buffered saline (PBS). At 20 h later, 500 μg DNP-BSA (Sigma-Aldrich) dissolved in 100 μL PBS containing 1% Evans blue dye was injected into the tail vein. Animals were euthanized 1.5 h after DNP-BSA challenge to assess Evan blue dye extravasation at the primed ears. Both ears were removed, minced, and placed into glass vials containing 0.5 mL dimethylformamide. Vials were then shaken at 500 rpm at 55°C for 3 to 5 h to extract dye from the tissue. The optical density of Evan blue was measured at 650 nm to reflect the degree of extravasation.

### Bioinformatic analysis of ChIP-seq and ATAC-seq data from the public domain.

To determine the epigenetic profile of the *FCER1A* locus, we queried the NCBI Gene Expression Omnibus (GEO) and retrieved the relevant data sets of GSE145542 and GSE145544 in TDF file format. The analyzed sequence data were visualized using IGV software.

### Statistics.

Graphs were generated by GraphPad Prism 7 (GraphPad Software, La Jolla, CA). Statistics were performed as described in the relevant figure legends with significance as *, *P* < 0.05; **, *P* < 0.01; ***, *P* < 0.001; and ****, *P* < 0.0001. Error bars represent the standard deviation (SD). Mean ± SD was derived from at least triplicate measurements. Differences between two samples were analyzed using unpaired *t* tests.

### Ethics statement.

The animal study was reviewed and approved by Committee on the Ethics of Animal Experiments of Tongji University (permit no. TJAA07920101).

### Data availability statement.

Sequencing data have been deposited in GEO under accession no. GSE199532.
