# Supplementary material for: A Co-Expressed Natural Antisense RNA FCER1A-AS Controls IgE-Dependent Immunity by Promoting Expression of FcεRIα
Source: Microbiol Spectr. 2023 May 8;11(3):e00733-23. doi: 10.1128/spectrum.00733-23 (PMC10269606; doi:10.1128/spectrum.00733-23)
Supplement: Supplemental file 1 — Supplemental material. Download spectrum.00733-23-s0001.pdf, PDF file, 1.5 MB [file spectrum.00733-23-s0001.pdf]

Fig.S1

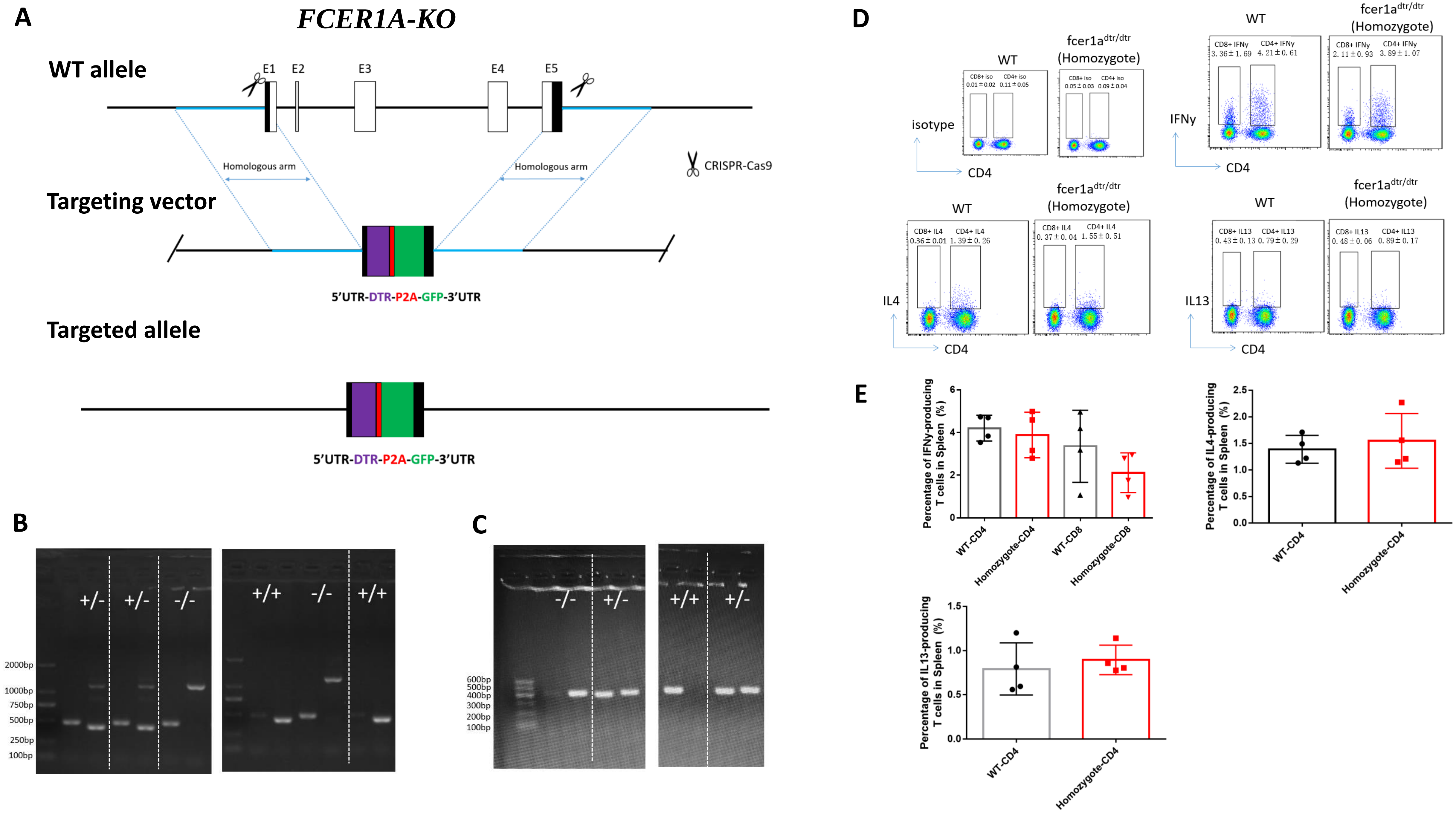

A
FCER1A-KO
WT allele
E1
E2
E3
E4
E5
Homologous arm
Homologous arm
CRISPR-Cas9
Targeting vector
5'UTR-DTR-P2A-GFP-3'UTR
Targeted allele
5'UTR-DTR-P2A-GFP-3'UTR
B
+/-
+/-
-/-
+/+
-/-
+/+
2000bp
1000bp
750bp
500bp
250bp
100bp
C
-/-
+/-
+/+
+/-
600bp
500bp
400bp
300bp
200bp
100bp
D
WT
fcer1a dtr/dtr (Homozygote)
isotype
CD4
CD8+ iso 0.01±0.02
CD4+ iso 0.11±0.05
CD8+ iso 0.05±0.03
CD4+ iso 0.09±0.04
IFNγ
CD8+ IFNγ 3.36±1.69
CD4+ IFNγ 4.21±0.61
CD8+ IFNγ 2.11±0.93
CD4+ IFNγ 3.89±1.07
IL4
CD8+ IL4 0.36±0.01
CD4+ IL4 1.39±0.26
CD8+ IL4 0.37±0.04
CD4+ IL4 1.55±0.51
IL13
CD8+ IL13 0.43±0.13
CD4+ IL13 0.79±0.29
CD8+ IL13 0.48±0.06
CD4+ IL13 0.89±0.17
E
Percentage of IFNγ-producing T cells in Spleen (%)
WT-CD4
Homozygote-CD4
WT-CD8
Homozygote-CD8
Percentage of IL4-producing T cells in Spleen (%)
WT-CD4
Homozygote-CD4
Percentage of IL13-producing T cells in Spleen (%)
WT-CD4
Homozygote-CD4

Figure S1：Extended information of construction of FCER1A-KO and genotyping for FCER1A$^{dtr/dtr}$ and FCER1A-KO

(A)Targeting strategy for FCER1A-KO. FCER1A-KO mice were generated via replacing the whole FCER1A region with DTR-P2A-GFP module without disruption of 5’UTR or 3’UTR by CRISPR-Cas9 strategy. Genotyping of FCER1A$^{dtr/dtr}$ and FCER1A-KO. (B) For FCER1A$^{dtr/dtr}$, PCR reaction was carried out using primer pairs Region2-F/Region2-R and Region2-F/Region1-R. (C) For FCER1A-KO, PCR reaction was carried out using primer pairs DTR2AGFP-F/DTR2AGFP-R and FCER1Ain-F/ FCER1Ain-R, Data shown represented one of three independent experiments. Th2 responses are unaffected in FcεRIα-deficiency mice of FCER1A$^{dtr/dtr}$.

IFN-γ–producing Th1 cells and IL-4/IL-13–producing Th2 cells were measured from Sj-infected splenocytes. A representative ICC staining (D) and the summarized results (E) were shown.

Fig.S2

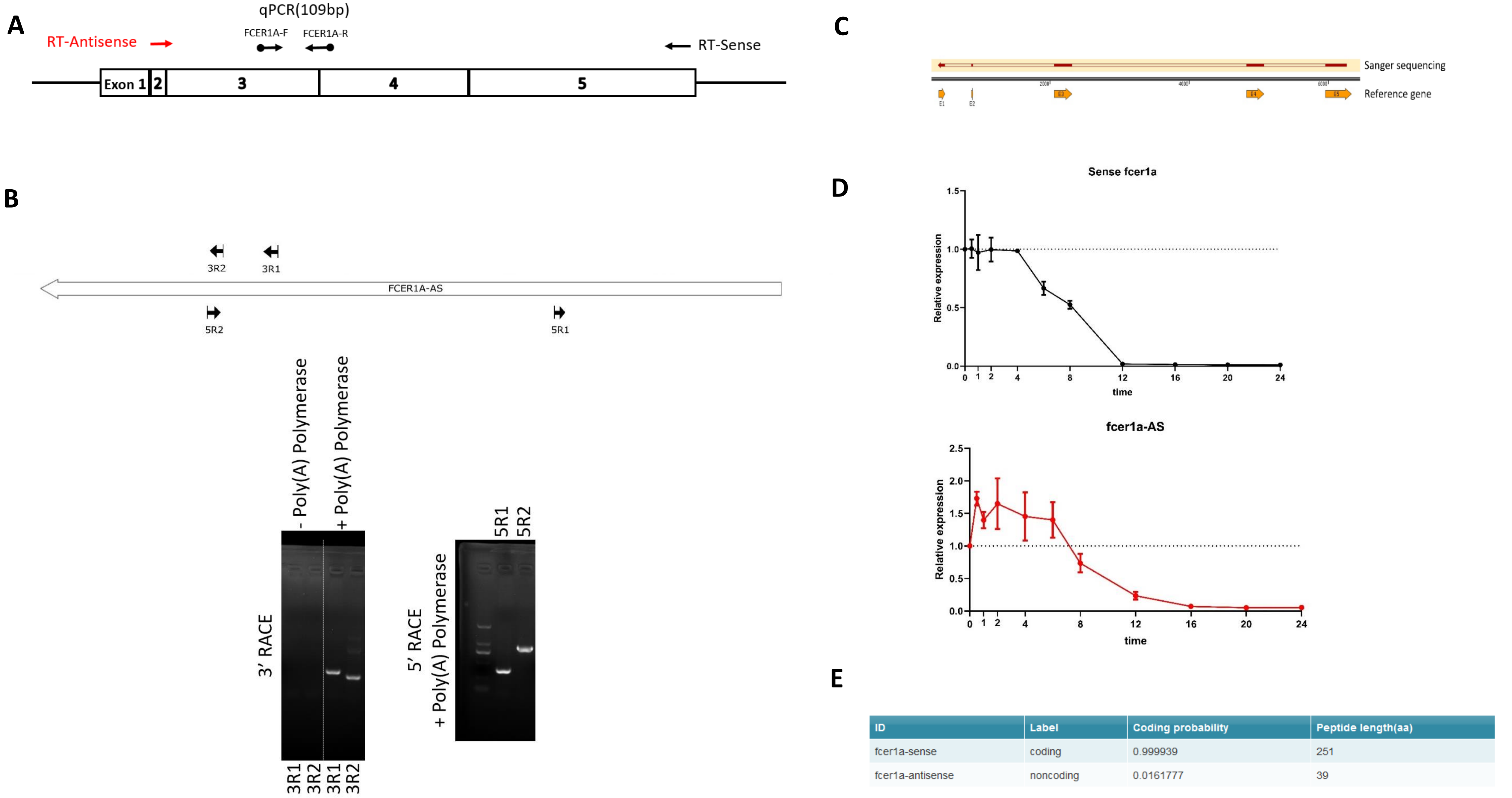

E

ID	Label	Coding probability	Peptide length(aa)
fcer1a-sense	coding	0.999939	251
fcer1a-antisense	noncoding	0.0161777	39

Figure S2：Extended information of FCER1A-AS

(A) Quantitative RT-PCR primers design for detection of sense (Black dotted-line) and antisense (red dotted-line) of FCER1A. RT primers for sense (black arrow; RT-WS) or antisense (red arrow; RT-Antisense) and qPCR primers (FCER1A-F/FCER1A-R). **(B)** Identification of the transcriptional starting site (TSS) and ending site of FCER1A-AS in MC/9 cell lines. 3’RACE fails unless polyA tails is added at 3’end of antisense RNA. Transcriptional ending site identified with 3’RACE assay in PolyA-added RNA. TSS identified with 5’RACE assay in PolyA-added RNA. Primers 3R1/3R2 and 5R1/5R2 indicated the primers used in RACE assay. **(C)** Align Sanger sequencing of FCER1A-AS to reference gene. **(D)** RNA stability of FCER1A-S and FCER1A-AS transcripts at different times points after actinomycin-D treatment. Amount of FCER1A-S and FCER1A-AS transcripts over time (at 0, 0.5, 1, 2, 4, 8, 12, 16, 20 and 24 h) after actinomycin-D treatment was measured by RT-PCR in MC/9 cells. Shown are expression levels at each tome point relative to time 0. Data are mean ± SD of three different experiments. **(E)** Bioinformatics analysis suggested that FCER1A-AS had no coding capability (http://cpc2.gao-lab.org/)

Fig.S3

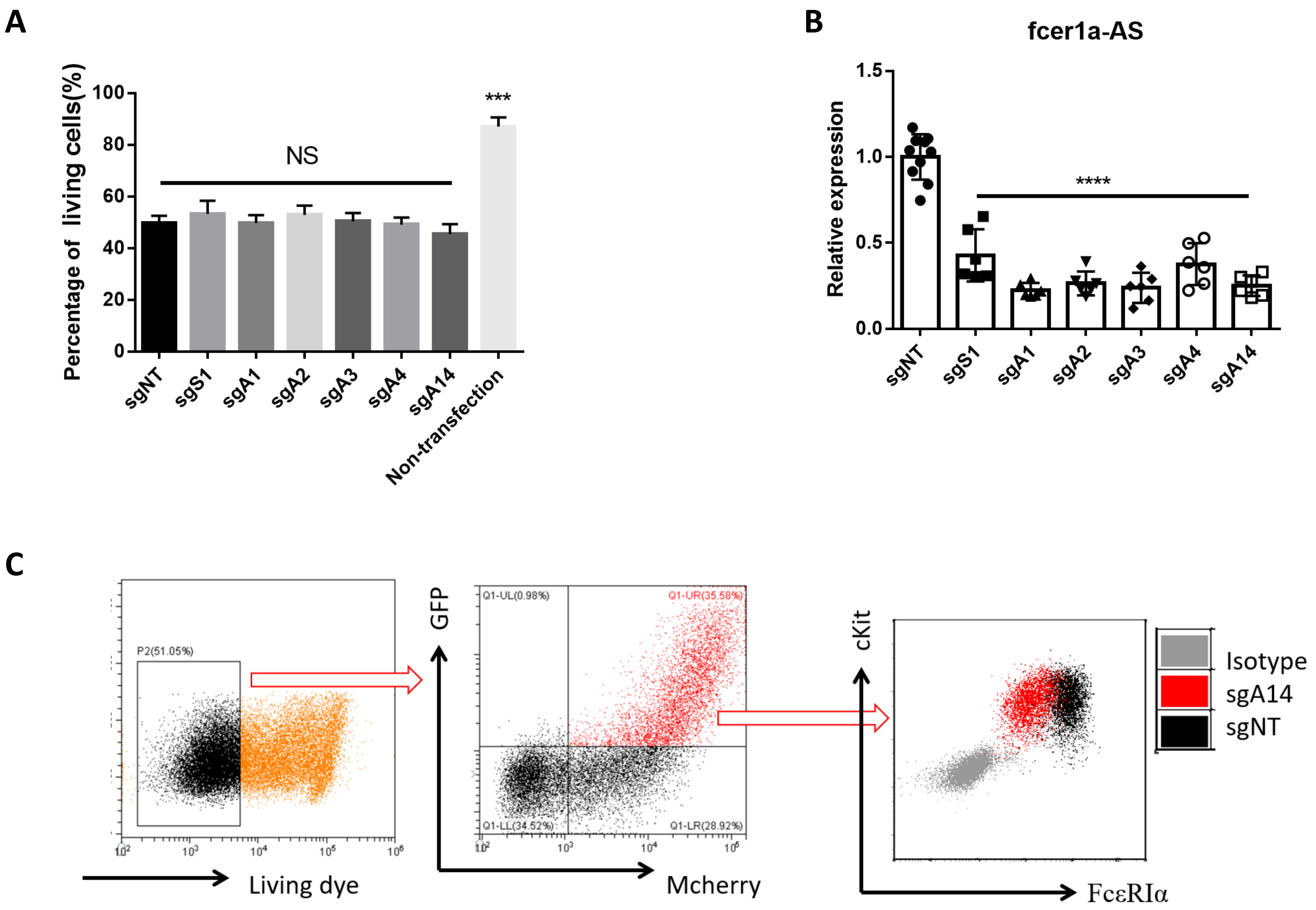
A
Percentage of living cells(%)
100
80
60
40
20
0
NS
***
sgNT
sgS1
sgA1
sgA2
sgA3
sgA4
sgA14
Non-transfection
B
fcer1a-AS
Relative expression
1.5
1.0
0.5
0.0
****
sgNT
sgS1
sgA1
sgA2
sgA3
sgA4
sgA14
C
P2(51.05%)
Living dye
GFP
Q1-UL(0.98%)
Q1-UR(35.58%)
Q1-LL(34.52%)
Q1-LR(28.92%)
Mcherry
cKit
FcεRIα
Isotype
sgA14
sgNT

Figure S3：Extended data for CRISPR/CasRx-mediated knockdown experiments in MC/9.

(A) Transfection of CasRX and sgRNA sgNT~sgA14 by electroporation did not affect viability differently. n=3 independent experiments. NS: no significant differences. **(B)** FCER1A-AS expression is successfully knocked down by FCER1A-AS-sgRNA (sgA) in MC/9 cells. Strand-specific quantitative RT-PCR of FCER1A-AS expression following FCER1A-AS-sgRNA (sgA) or FCER1A-S-sgRNAs (sgS) transfection. Relative expression indicates the ratio relative to control sgNT. Data are mean ± SD of six different experiments. ****$P < 0.0001$. **(C)** Gating Strategy for CasRX/sgRNA co-transfected cells.The GFP-positive (CasRX transfected) and Mcherry-positive (sgRNA transfected) population was selected on viable cells. In the case shown here, the cell surface expression of FcεRIα was markedly reduced by sgRNA A14 targeting onto FCER1A-AS comparing to non-targeting sgRNA NT control.

Fig.S4

A

±	score	% div.	% del.	% ins.	query sequence	position in query- begin	end	(left)	C +	matching repeat	repeat class/family	-position in repeat- (left) begin	end end	begin (left)	linkage id/graphic
+	181	34.7	5.0	0.8	s	303	421	(1970)	C	MIR3	SINE/MIR	(15)	193	70	1
+	48	0.0	0.0	0.0	s	882	925	(1466)	+	(AAAAC)n	Simple_repeat	1	44	(0)	2

```
  s                       303 AAAGTGTAAGA--TTAAGGAGACTCAAGAAACCACCTTGTCCAATCCCTT 350
                               i i   v   -- vi i i   iiv   i i  i  v      i   i
C MIR3#SINE/MIR           193 AGAATGTCAGAGCTGGAAGGGACCTTAGAGATCATCTAGTCCAACCCCCT 144

  s                       351 C-TCATACAA--GAATAAACAGAATCTCAAAGACTTTCAAGTAGCTTG-G 396
                               - iv    i--  iv    v  iv i  i   -vv v    ii    -v
C MIR3#SINE/MIR           143 CATTTTACAGATGAGGAAACTGAGGCCCAGAGA-GGTGAAGTGACTTGCC 95

  s                       397 CACAGTCCAGCACCCAGTGAGAAGC 421
                                vi   vvi  v ?   v  vi
C MIR3#SINE/MIR            94 CAAGGTCACACAGCNAGTTAGTGGC 70


  s                       882 AAAACAAAACAAAACAAAACAAAACAAAACAAAACAAAACAAAA 925

 (AAAAC)n#Simp              1 AAAACAAAACAAAACAAAACAAAACAAAACAAAACAAAACAAAA 44
```

B

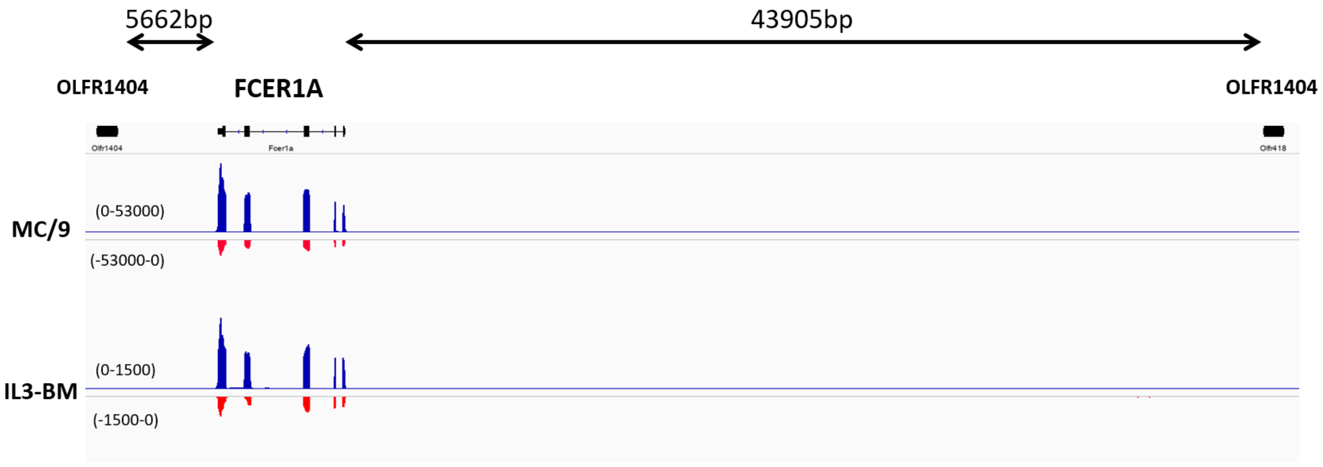

Figure S4: Putative transcription initiation analysis for FACERIA-AS. (A) Transposon MIR element and simple repeat sequence is spread within this promoter region of FCER1A-AS. **(B)**The transcription of FCERIA-AS is not due to extension of sense gene transcription of neighboring gene located 3’ down stream of FCERIA.

Table S1: Primers used in this study

For genotyping		5' > 3'
FCER1A$^{dtr/dtr}$	Region1-reserve	AGCAGTCAGCTCATTCCTTAACTT
	Region2-forward	CCATTCTAGCTTACAAATGCAAGTA
	Region2-reserve	TGTGGCTTGGAGGATAAAGTGAC
FCER1A-KO	fcer1a(e3)-f	ACTCCTTGCATTTGTGCCCT
	fcer1a(e3)-R	AACGGTGGCACTCACAATGA
	DTR2AGFP-f	TCGTGGGGCTTCTCATGTTT
	DTR2AGFP-R	GACTTGAAGAAGTCGTGCTGC

5' > 3'

RT-Sense	AGGCAGTGTTTATTGAGTATTTGCT
RT-Antisense	CTCTTGATGTCATTCTGACAG
RT-WS	AGGCAGTGTTTATTGAGTATTTGCT
PCR-WS1	ATTGTGAGTGCCACCGTTCA
PCR-WS2	CTCTTGATGTCATTCTGACAG
RT-WA1	GGAACTAATGTCATTGCTGAAGAAG
RT-WA2	ACAAATGCAAGTATTATTGG
RT-WA3	TGGCTGCTCCTTCAGACATC
RT-WA4	ATTGTGAGTGCCACCGTTCA
PCR-WA	AGGCAGTGTTTATTGAGTATTTGCT
RT-MIA1	GGAACTAATGTCATTGCTGAAGAAG
RT-MIA2	CACTGGATCTACGGACCAGC
RT-MIA3	ACAAATGCAAGTATTATTGG
RT-MIA4	TGGCTGCTCCTTCAGACATC
PCR-MIA	AGGCAGTGTTTATTGAGTATTTGCT
FCER1A-F	ATTGTGAGTGCCACCGTTCA
FCER1A-R	GAAGGAGCAGCCAATCTTGC

DTR-p2A-GFP-F	ATCGTGGGGCTTCTCATGTTT
DTR-p2A-GFP-R	CTTGTGGCCGTTTACGTCG
Exon-p2A-DTR-f	GTTATTGCTCTCAACCGAAG
Exon-p2A-DTR-R	AGAAGGAGCTTCAGCACCAC
DTR-F	CACTGGATCTACGGACCAGC
DTR-R	TTTTCCCGTGCTCCTCCTTG
Gata2-F	TGCACAATGTTAACAGGCCAC
Gata2-R	CCTCGAAACATTCAGCCCCT
CD200R3-F(q)	GATTGAGATTATCACTCCAGTGTCT
CD200R3-R(q)	CTGATCTGCTGCAACTCTGCT
5R1	ACCGAAGAACAGTTCAAA
5R2	AGTGACCCTTTCCTGCTATGG
3R1	TCACAATGACCAAATGTGAAGA
3R2	GTTCCCATAGCAGGAAAGGGT

Table S2: sgRNA sequence

	5’ > 3’
Sense sg1	GCACAGCTGGGCAGACCTTCCAGTGACCAT
AS sg1	GATTCTGTTTGCTGTGGACACGGGGTTATT
AS sg2	GAAAATACAAGAAAGTTGAAACCGAACTCC
AS sg3	AGTCTGTTCCACAGGAATTGCATAAATGCT
AS sg4	CTGGTTATGTAACATGCAATAAATAGCAAA
AS sg 1-4	GATTCTGTTTGCTGTGGACACGGGGTTATTCAAGTAAACCCCTACCAACTGGTCGGGGTTTGAAAC<br>GAAAATACAAGAAAGTTGAAACCGAACTCCCAAGTAAACCCCTACCAACTGGTCGGGGTTTGAAAC<br>AGTCTGTTCCACAGGAATTGCATAAATGCTCAAGTAAACCCCTACCAACTGGTCGGGGTTTGAAACC<br>TGGTTATGTAACATGCAATAAATAGCAAA

Table S3: Fluorescence-labeled antibodies and reagents for live cell detection

APC-labeled anti-mouse FcεRIa (MAR-1; BioLegend)
PE-cy7-labeled anti-mouse CD49b (DX5; eBioscience)
PE-labeled anti-mouse ckit(Ba13, BD Pharmingen)
PE-labeled anti-mouse CD200R3 (ACK2, BD Pharmingen)
FITC-labeled anti-mouse CD3ε (145-2C11; BioLegend)
Percp-Cy5.5–labeled anti-mouse B220 (RA3-6B2; BioLegend)
PE-labeled anti-mouse CD4 (GK1.5; Biolegend)
APC-labeled anti-mouse IFN-γ (XMG1.2; Biolegend)
APC-labeled anti-mouse IL-4 (11B11; Biolegend)
APC-labeled anti-mouse IL-13 (W17010B; Biolegend)
Fixable viability dye eFluor 780 (eBioscience)
